# Challenges for the development of alternative low-cost ventilators during COVID-19 pandemic in Brazil

**DOI:** 10.5935/0103-507X.20200075

**Published:** 2020

**Authors:** Erica Aranha Suzumura, Ana Denise Zazula, Henrique Takachi Moriya, Cristina Quemelo Adami Fais, Alembert Lino Alvarado, Alexandre Biasi Cavalcanti, Ricardo Goulart Rodrigues

**Affiliations:** 1 Preventive Medicine Department, Faculdade de Medicina, Universidade de São Paulo - São Paulo (SP), Brazil.; 2 Faculdade de Medicina, Pontifícia Universidade Católica do Paraná - Curitiba (PR), Brazil.; 3 Biomedical Engineering Laboratory, Escola Politécnica, Universidade de São Paulo - São Paulo (SP), Brazil.; 4 Device Technology Management, Gerência- Geral de Tecnologia em Produtos para a Saúde, 3ª Diretoria, Agência Nacional de Vigilância Sanitária -Brasília (DF), Brazil.; 5 Research Institute, HCor - Hospital do Coração, São Paulo - (SP), Brazil.; 6 Intensive Care Service, Hospital do Servidor Público Estadual “Francisco Morato de Oliveira” - São Paulo (SP), Brazil.

**Keywords:** Ventilators, mechanical, Respiration, artificial, Positive-pressure respiration, COVID-19, Severe acute respiratory syndrome, Biomedical engineering, Brazil, Ventiladores mecânicos, Respiração artificial, Respiração com pressão positiva, COVID-19, Síndrome respiratória aguda grave, Engenharia biomédica, Brasil

## Abstract

The COVID-19 pandemic has brought concerns to managers, healthcare professionals, and the general population related to the potential mechanical ventilators’ shortage for severely ill patients. In Brazil, there are several initiatives aimed at producing alternative ventilators to cover this gap. To assist the teams that work in these initiatives, we provide a discussion of some basic concepts on physiology and respiratory mechanics, commonly used mechanical ventilation terms, the differences between triggering and cycling, the basic ventilation modes and other relevant aspects, such as mechanisms of ventilator-induced lung injury, respiratory drive, airway heating and humidification, cross-contamination risks, and aerosol dissemination. After the prototype development phase, preclinical bench-tests and animal model trials are needed to determine the safety and performance of the ventilator, following the minimum technical requirements. Next, it is mandatory going through the regulatory procedures as required by the Brazilian Health Regulatory Agency (*Agência Nacional de Vigilância Sanitária* - ANVISA). The manufacturing company should be appropriately registered by ANVISA, which also must be notified about the conduction of clinical trials, following the research protocol approval by the Research Ethics Committee. The registration requisition of the ventilator with ANVISA should include a dossier containing the information described in this paper, which is not intended to cover all related matters but to provide guidance on the required procedures.

## Abstract (pt)

A pandemia por COVID-19 tem deixado os gestores, os profissionais de saúde e a população preocupados com a potencial escassez de ventiladores pulmonares para suporte de pacientes graves. No Brasil, há diversas iniciativas com o intuito de produzir ventiladores alternativos para ajudar a suprir essa demanda. Para auxiliar as equipes que atuam nessas iniciativas, são expostos alguns conceitos básicos sobre fisiologia e mecânica respiratória, os termos comumente utilizados no contexto da ventilação mecânica, as fases do ciclo ventilatório, as diferenças entre disparo e ciclagem, os modos ventilatórios básicos e outros aspectos relevantes, como mecanismos de lesão pulmonar induzida pela ventilação mecânica, pacientes com *drive* respiratório, necessidade de umidificação de vias aéreas, risco de contaminação cruzada e disseminação de aerossóis. Após a fase de desenvolvimento de protótipo, são necessários testes pré-clínicos de bancada e em modelos animais, a fim de determinar a segurança e o desempenho dos equipamentos, seguindo requisitos técnicos mínimos exigidos. Então, é imprescindível passar pelo processo regulatório exigido pela Agência Nacional de Vigilância Sanitária (ANVISA). A empresa responsável pela fabricação do equipamento deve estar regularizada junto à ANVISA, que também deve ser notificada da condução dos testes clínicos em humanos, seguindo protocolo de pesquisa aprovado pelo Comitê de Ética em Pesquisa. O registro do ventilador junto à ANVISA deve ser acompanhado de um dossiê, composto por documentos e informações detalhadas neste artigo, que não tem o propósito de esgotar o assunto, mas de nortear os procedimentos necessários.

## INTRODUCTION

In December 2019, several cases of respiratory syndrome caused by a new coronavirus were reported.^(1)^ Subsequently, the virus called severe acute respiratory syndrome coronavirus 2 (SARS-CoV-2) and the disease associated with it, COVID-19, spread worldwide. The first case in Brazil was confirmed on February 26^th^, 2020, and since then, the number of new cases and deaths from COVID-19 have been on a rise in the country.

Around 20% of patients with COVID-19 require hospital admission, mostly due to respiratory distress.^(2)^ From these, about 15% require intensive care unit (ICU) admissions to receive support for organ dysfunctions such as mechanical ventilation for respiratory failure.^(2)^

Given the rapidly progressing number of patients with severe COVID-19 requiring ICU admission and the use of mechanical ventilation, in addition to patients who require this type of support for other reasons, there is a growing concern regarding possible mechanical ventilators shortage. Therefore, physicians and other healthcare professionals may face a difficult decision: who out those critically ill patients will be placed on mechanical ventilation if there is not enough for all?^(3-5)^

National and international companies report that the number of orders for mechanical ventilators skyrocketed since pandemic was declared by the World Health Organization on March 11^th^, 2020. The high demand for mechanical ventilators, associated with the high complexity in manufacturing and limited production capacity of the industry, has delayed and rendered difficult delivering the requested machines.

Mechanical ventilators are medical devices that have complex technology with volumetric and pressometric control. They feature digital manometer for pressure control and real-time pressure, volume and flow curves. The available settings and functions allow operation in different ventilation modes. These devices have standard alarms (blackout, oxygen drop, low battery, inverse inspiratory/expiratory (I:E) ratio, and disconnection from the patient) and settable alarms (maximum peak inspiratory pressure, volume, respiratory rate - RR, positive end-expiratory pressure - PEEP and apnea) that must be adjusted according to the individual needs of each patient. Some mechanical ventilators have additional functions, such as compensation for tube resistance, for air leakage and circuit compliance, or noninvasive ventilation mode. Also, overpressure valves are needed on the gas supply and ventilator systems. The development of a mechanical ventilator involves numerous steps and tests, which can require commercial manufacturers to expend up to 2 years to complete.

Thousands of experts, entrepreneurs, and volunteers worldwide are working to create alternative ventilators in an attempt to prevent COVID-19 patients’ deaths for ventilator shortage. In Brazil, there are several initiatives focused on rapidly producing large-scale alternative low-cost ventilators to be used in these patients.

Prototypes based on automation of the artificial manual breathing unit (AMBU), on Boussignac valve, and on bellows are currently being developed, as well as reproductions of the Takaoka mini pneumatic ventilator, and attempts to develop more complex mechanical ventilators with microprocessor technology.^(6)^ These prototypes would have a life-saving potential for patients who would have no other chance for survival if no conventional mechanical ventilator is available. However, a medical device aimed at saving lives, if not appropriately developed to provide the required functions, can cause irreversible or fatal harm.

## THE CHALLENGE FOR THE DEVELOPERS

One of the challenges for alternative ventilator developers who have not a health science background, is the lack of expertise in physiology, respiratory mechanics, and ventilator-induced lung injury mechanisms.

Biomedical engineering courses emphasize the need for acquiring information related to medical concepts, the reason why many classes are given by healthcare professionals.^(7)^ The detailed knowledge on the available ventilation modes is the best way to get safe and effective mechanical ventilation^(8)^ as well to develop medical devices that meet the patients’ needs. If not, the medical device may become a mere potentially lethal air pumping device. In this sense, some basic concepts are presented, and it is still essential that the teams that propose to develop alternative ventilators involve professionals with respiratory pathophysiology knowledge and expertise in management of patients in mechanical ventilation.

### Basic respiratory physiology

Breathing aims to provide oxygen to and remove carbon dioxide produced by the body tissues.^(9)^ Under normal conditions, inspiration is achieved by expanding the chest, which occurs by contraction of inspiratory muscles (diaphragm and external intercostal muscles). This chest expansion decreases the pressure within the respiratory system. Air enters the lungs when the alveolar pressure is lower than the pressure at the opening of the upper airways.^(9)^

Normal expiration is passive and requires no work. The muscles relax, the diaphragm rises, returning to the resting position, the chest volume is reduced, and the air flows out the alveoli, exiting through the upper airways.^(9,10)^

**Figure 1:**
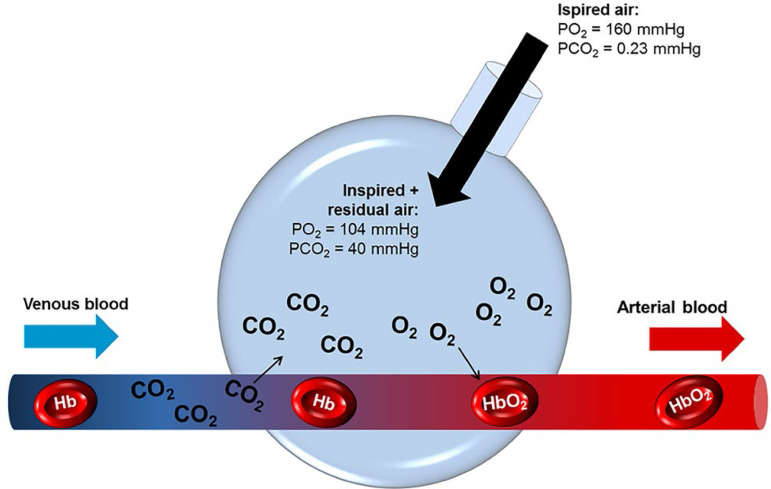
Diffusion gas exchange between alveoli and pulmonary capillary vessels. PO_2_ - partial oxygen pressure; PCO_2_ - partial carbon dioxide pressure; CO_2_ - carbon dioxide; O_2_ - oxygen; Hb - hemoglobin.

The gas exchange between the alveolar air and the blood present in the pulmonary capillaries surrounding the alveoli occurs through diffusion.^(9,11)^ The higher oxygen concentration in the alveoli causes this gas to diffuse into the pulmonary capillary, while the higher carbon dioxide concentration in capillary blood, causes carbon dioxide to diffuse into the alveoli.^(9,11)^ The alveolar-capillary membrane, where this exchange takes place, is extremely thin and delicate. After gas exchange, blood circulates through the body, transporting oxygen to be delivered to the cells, and capturing carbon dioxide produced by them (Figure 1).^(9)^

During mechanical ventilation, the pressure gradients leading the air into the lungs are obtained through positive pressures applied to the opening of the airways, literally pushing air into the respiratory system. This mechanism is opposed to the physiological spontaneous breathing. Therefore, mechanical ventilation should be carefully adjusted to avoid harmful effects.

### Mechanical ventilation basics

#### Mechanical ventilation terminology

In mechanical ventilation, there are specific concepts and terminologies. The commonly used terms are:^(12,13)^

- Tidal volume (TV): the volume of air delivered into the lungs with each breath by the mechanical ventilator. The volumes involved in ventilation are usually expressed in liters (L) or milliliters (mL).
- Respiratory rate (RR): the number of breaths in one minute, which may be either determined by the ventilator, or by the patient, or both.
- Minute ventilation (V_E_): the total air volume mobilized in one minute. Therefore, V_E_ is the product of TV times RR (V_E_ = TV * RR). It can be calculated for either expiration or inspiration.
- Peak pressure (P_peak_): the highest level of pressure in the respiratory system generated by the TV during inhalation. The pressures involved in ventilation are usually expressed in centimeters of water (cmH_2_O).
- Plateau pressure (P_plateau_): the pressure of the pulmonary parenchyma distension. Its assessment requires a minimum 2-minutes respiratory pause (occlusion of the respiratory system at the end of the inspiration).
- Peak inspiratory flow: the maximum flow at which a set TV breath is delivered by the ventilator. The flow can be either fixed during the inspiration (e.g. a constant flow wave or “squared” on volume control ventilation mode) or variable (e.g. a decelerating flow wave on volume control ventilation mode, or pressure control ventilation and pressure support ventilation modes).
- PEEP: a positive pressure maintained within the lungs (alveolar pressure) at the end of expiration, aimed at preventing alveolar closure at the end of expiration.
- Fraction of inspired oxygen (FiO_2_): the oxygen concentration in the inspired air. It may range from 0.21 to 1.0 (21% to 100%).
- Sensitivity: reflects the patient’s drive to start an inspiration. The detection may occur either by monitoring the pressure, the flow, or a neuronal-related signal.
- Inspiratory/expiratory (I:E) ratio: the ratio between the inspiratory time over the expiratory time. It may range according to the patient´s demand and mechanical ventilation objective, usually ranging between 1:1 and 1:3.
- Ventilator breathing circuit: the set of tubes and connectors leaving the ventilator and taking the air to the patient, and from the patient to the expiratory valve.

#### Phases of breath during mechanical ventilation

**Figure 2:**
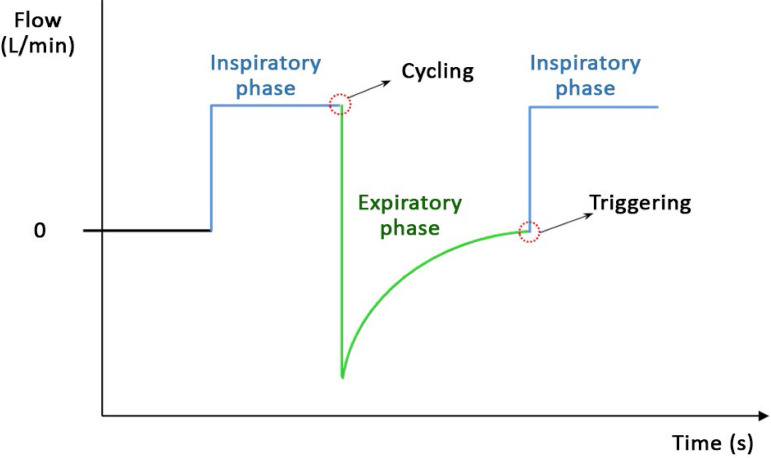
Phases of breath during mechanical ventilation (flow curve during continuous mandatory ventilation using volume control).

During positive pressure mechanical ventilation, a breath has four phases (Figure 2):^(14,15)^

1. Inspiratory phase: this phase begins once the flow valve is open and a given volume of air is provided, overcoming the elastic and resistive loads of the respiratory system, and the lungs are inflated.
2. Cycling: the change from the inspiratory to the expiratory phase (end of inspiration).
3. Expiratory phase: this phase begins once the expiratory valve is open, and the lungs are passively deflated.
4. Triggering: the change from the expiratory to the inspiratory phase (start of inspiration).

#### Cycling

As previously described, it is the change from the inspiratory to the expiratory phase. There are five types of cycling:^(8,12,16)^

1. Pressure cycling: the inspiration is interrupted when a given pressure threshold is reached. This is the Bird Mark 7 ventilator’s mechanism of cycling. The inspiratory time and TV are variable, depending on pulmonary compliance and resistance.
2. Time cycling: the inspiration is interrupted when a predetermined time is reached. This mechanism is used on the pressure control ventilation (PCV) mode and volume control ventilation (VCV) mode with inspiratory pause. The inspiratory time is predetermined allowing no patient interaction. On PCV mode, the pressure is set, and the TV depends on pulmonary compliance and resistance. On VCV, the TV is set, and the pressure depends on pulmonary compliance and resistance.
3. Volume cycling: the inspiration is interrupted when a predetermined TV is reached. It is used on the VCV mode without inspiratory pause. The volume is ensured, regardless of pulmonary compliance and resistance, however, the pressures may be variable.
4. Flow cycling: the inspiration is interrupted when the flow decreases to predetermined levels (e.g.: 25% of the peak inspiratory flow). It is the mechanism used on the pressure support ventilation (PSV) mode. The TV is variable and depends on pulmonary compliance and resistance.
5. Neural cycling: occurs when the respiratory center in the brainstem interrupts inspiration. This mechanism is based on the use of the signal obtained from the electric activity of diaphragm (EAdi) for ventilation control, and is exclusive for the neurally adjusted ventilatory assist (NAVA) mode. The EAdi is a direct representation of the central respiratory drive and reflects the duration and intensity with which the patient wishes to ventilate. For the recording of the EAdi signal, the system uses electrodes embedded in the distal part of an esophageal catheter.

#### Triggering

During conventional mechanical ventilation, the ventilator triggering may be either programmed or by the patients’ inspiratory effort. The four most common types of triggering are:^(12,15,16)^

1. Time triggering: occurs when the patient shows no inspiratory effort to trigger the ventilator, e.g., during deep sedation or neuromuscular blockade, or when there is no central nervous system drive. The ventilator triggering is based on the RR setting programmed, and the ventilation is delivered independent of the patient’s spontaneous efforts.
2. Pressure triggering: when the system pressure becomes negative, indicating a patient’s inspiratory effort, the flow valve is open, triggering an inspiratory phase.
3. Flow triggering: analogous to the pressure triggering, but in this case, the device can detect an inspiratory effort through a variation of flow in the system.
4. Neural triggering: occurs when the respiratory center in the brainstem triggers an inspiration. This triggering mechanism is based on EAdi signal during NAVA. As the triggering is synchronized with diaphragm excitation, it reduces the ventilator’s response time, favoring the neuro-ventilatory coupling.

#### Ventilation modes

Ventilation modes refers to the method of providing and controlling the breaths. Several modes are available, including synchronized intermittent mandatory ventilation (SIMV), but the basic ventilation modes in the context of alternative ventilators are:^(14,15,17)^

1. Continuous mandatory ventilation (CMV): the ventilator only provides mandatory breaths, using either volume (VCV) or pressure (PCV) control, based on the programmed RR. The ventilator settings are adjusted according to the selected mode. No sensitivity adjustments are made, because, in this mode, patient´s effort is not allowed to trigger the ventilator. Its main disadvantage is that, even though the patient exhibits respiratory effort, the ventilator does not allow triggering synchronized with the patient’s effort. Patients under this condition can develop respiratory muscle fatigue and severe adverse events, such as barotrauma. In clinical practice, CMV mode is not used.
  1.1. VCV: TV is fixed and set by the operator. The other adjustable parameters are PEEP, inspiratory pause, inspiratory flow or inspiratory time (which determines the flow), RR, and FiO_2_.
  1.2. PCV: the inspiratory pressure or driving pressure (pressure difference above the PEEP) is fixed and set by the operator. The other adjustable parameters are PEEP, inspiratory time, rise time (determines the time at which the ventilator achieves a target airway pressure), RR and FiO_2_ (Figure 3).
    **Figure 3:**
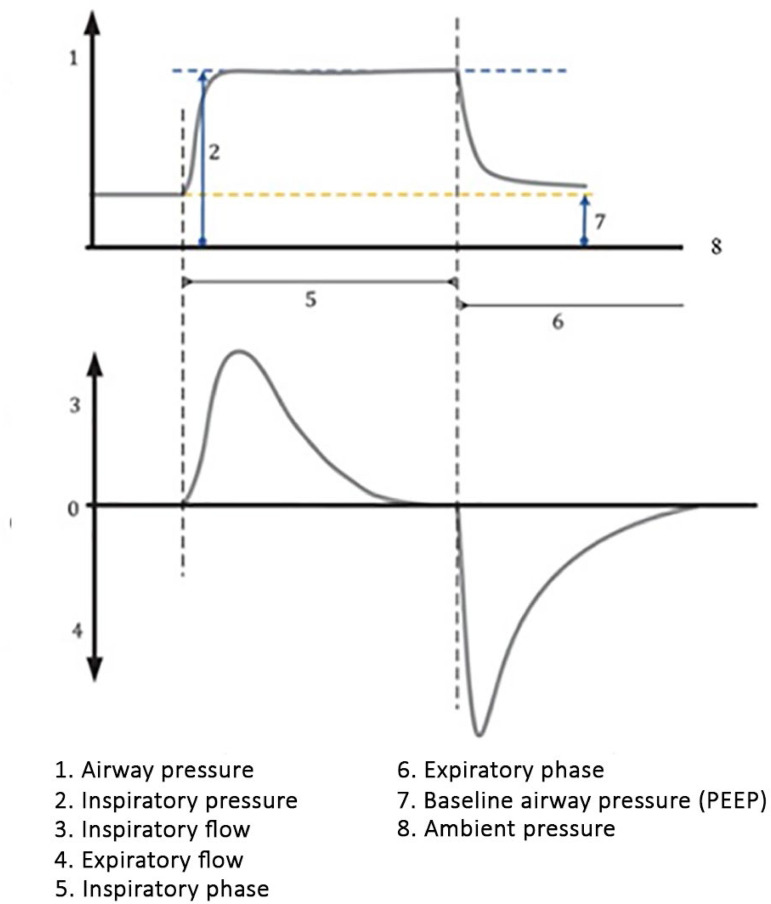
Pressure and flow curves during continuous mandatory ventilation using pressure control mode. PEEP - positive end-expiratory pressure.

Source: Adapted from: The International Organization for Standardization (ISO). ISO 19223:2019(en) Lung ventilators and related equipment - Vocabulary and semantics. [cited 2020 May 19]. Available from: https://www.iso.org/obp/ui#iso:std:iso:19223:ed-1:v1:en:ed%201:v1.^(17)^
2. Assist/control (A/C) ventilation: the ventilator provides mandatory and assisted breaths, using either volume (VCV) or pressure (PCV) control. If the patient shows inspiratory effort, the flow valve will be opened, allowing an assisted breath according to the ventilator´s settings. If the patient ceases inspiratory effort, then the ventilator assumes the RR as set and triggers mandatory ventilations.
  2.1. VCV: as with CMV, the TV is set by the operator. In addition to other parameters, the sensitivity for triggering assisted breaths is adjustable (Figure 4).
    **Figure 4:**
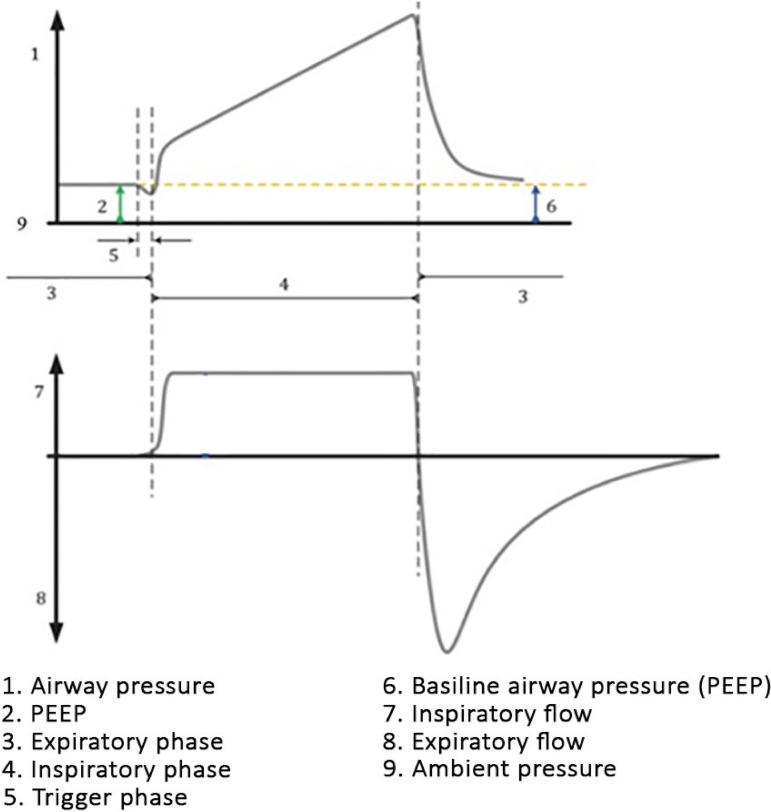
Pressure and flow curves during assist/control ventilation using volume control mode. PEEP - positive end-expiratory pressure.

Source: Adapted from: The International Organization for Standardization (ISO). ISO 19223:2019(en) Lung ventilators and related equipment - Vocabulary and semantics. [cited 2020 May 19]. Available from: https://www.iso.org/obp/ui#iso:std:iso:19223:ed-1:v1:en:ed%201:v1.^(17)^
  2.2. PCV: as with CMV, the inspiratory pressure is set by the operator. In addition to other parameters, the sensitivity for triggering assisted breaths is adjustable (Figure 5).
    **Figure 5:**
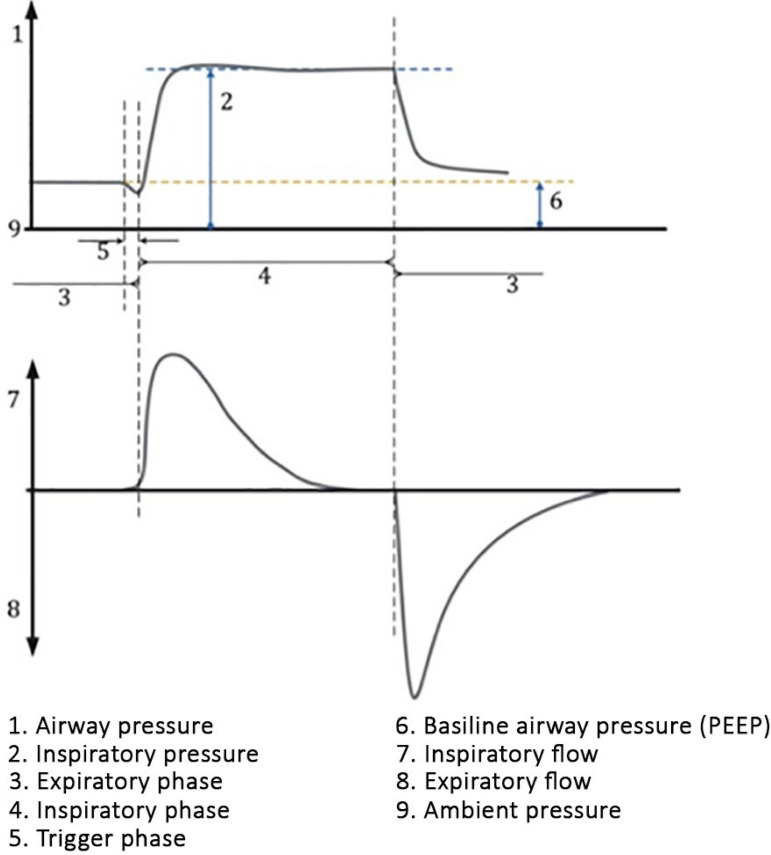
Pressure and flow curves during assist/control ventilation using pressure control ventilation. PEEP - positive end-expiratory pressure.

Source: Adapted from: The International Organization for Standardization (ISO). ISO 19223:2019(en) Lung ventilators and related equipment - Vocabulary and semantics. [cited 2020 May 19]. Available from https://www.iso.org/obp/ui#iso:std:iso:19223:ed-1:v1:en:ed%201:v1.^(17)^
3. PSV: it is considered a spontaneous ventilation mode. Every breath is started and maintained by the patient, and the ventilator delivers support with the preset pressure (pressure difference above the PEEP) during the inspiratory phase. Cycling takes place when the flow decreases to a given percent of the peak inspiratory flow, which can be automatic (usually 25%), or in modern ventilators, it can be set (between 5% and 80%). When this flow decrease is detected, the ventilator interrupts the pressure support and allows cycling. The parameters to be set are the pressure support, rise time, PEEP, sensitivity, termination criterion (percent of peak inspiratory flow drop for cycling, when available), and FiO_2_.

### Patients with respiratory drive

Some of the alternative ventilator projects do not take into account patients who show inspiratory effort (respiratory drive). Except for exceptional conditions, most of the patients in clinical practice present inspiratory effort, and commercial mechanical ventilators provide a synchronized “reinforcement” to spontaneous breaths. The time-to-response to inspiratory effort has a significant influence on patient/ventilator synchrony and intrinsically depends on the type of sensor employed.^(18,19)^

When the mechanical ventilator only provides CMV, patients with respiratory drive, either under mild or no sedation, or those in weaning from mechanical ventilation are at great risk of severe adverse events, such as ventilation-induced lung injury, barotrauma and worsing of gas exchange, making it difficult to regain autonomous breathing without devices. Therefore, alternative ventilators not allowing synchronous breathing should not be used in long term support of critically ill patients.

### Humidification

During spontaneous breathing, inhaled air is humidified and heated during its passage through the airway, reaching the alveoli appropriately saturated with water and at body temperature (100% relative humidity and 37ºC). Patients undergoing invasive mechanical ventilation lose natural inhaled gas heating and humidification mechanisms, which increases the risk of complications such as changes in mucociliary clearance, secretions thickening, and tracheal tube clogging. These complications may lead to hypothermia, hypoxemia, atelectasis and lung inflammation.^(20-22)^ Therefore, during invasive mechanical ventilation external humidifiers are needed to compensate for the lack of natural heating and humidification mechanisms when the upper airway is bypassed.^(23,24)^ Ventilators development teams should consider the types of humidification and heating devices to be used. Active heated humidifiers, placed in the inspiratory limb of the ventilator circuit, act by allowing air passage inside a heated water reservoir before traveling to the patient’s airway. Condensation of water vapor inside the ventilator circuit requires frequent disconnections to remove the accumulated liquid, causing aerosols dissemination and environmental contamination. Hence, active heated humidifiers are not recommended for patients with COVID-19. The alternative ventilator should allow the connection to a heat and moisture exchanger (HME) passive humidification system to avoid such disconnections.

### Cross-contamination

To prevent cross-contamination, some components of alternative ventilators should be sterilizable, such as the expiratory valve, the inspiratory valve, and the flow sensor. This is fundamental for choosing components manufacture materials and their fixating methods, allowing removal for appropriate cleaning.

It is also essential that the ventilators allow the connection to high-efficiency particulate arrestance (HEPA) filters at the ventilator circuit expiratory exit, to prevent aerosol dissemination and environmental contamination.

### Ventilation-induced lung injury

Mechanical ventilation is an essential part of therapy in patients with severe respiratory failure. However, mechanical ventilation itself may cause alveolar injury or worsen an established disease.^(25)^ The possible injury mechanisms include barotrauma, volutrauma, biotrauma, and atelectrauma.^(26)^

Barotrauma indicates lung injury attributed to the application of high pressures, and its gross presentations being pneumothorax, pneumomediastinum, subcutaneous emphysema, and gas embolism.^(27)^ Volutrauma means lung injury induced by high tidal volumes.^(28)^ Biotrauma occurs when excessive pressures and volumes are used, even if not enough to cause barotrauma and volutrauma, leading to proinflammatory cytokines release, leukocytes recruitment, and inflammation.^(29)^ Prolonged and exacerbated biotrauma can lead to multiple organ dysfunction syndrome increasing the risk of death.^(29)^

Atelectrauma is the lung injury caused by cyclic opening and closure of the alveoli.^(30)^ A mathematical model suggests that the pressures acting at the interface between open alveoli and closed units can be as high as 140cmH_2_O, even with airway pressure of 30cmH_2_O.^(31)^

The protective mechanical ventilation strategy is based on avoiding such phenomena.^(32,33)^ Barotrauma, volutrauma, and biotrauma can be avoided by ventilation with low volumes and pressures, appropriately individualized for each patient. Atelectrauma can be avoided with the use of PEEP, aiming to keep the alveoli open, also individualized to prevent untoward alveolar hyperdistention.

Therefore, it is not advisable that experimental ventilators, particularly those based on AMBU, to use only standard mechanical safety valves (also known as ‘pop-off valve’) limiting P_peak_ to 60cmH_2_O. Some of these valves can be accidentally locked, with no alert to the assisting team, which allows the pressure in the respiratory system to increase considerably, potentially causing severe lung injuries.

## MINIMUM TECHNICAL REQUIREMENTS

On March 20^th^, 2020, the Brazilian Health Regulatory Agency (*Agência Nacional de Vigilância Sanitária* - ANVISA) published the Resolution of the Collegiate Board (RDC) 349, defining criteria, and extraordinary and temporary procedures for dealing with approval requests of new mechanical ventilators identified as strategic, due to the international public health SARS-CoV-2 emergency. This RDC is valid for 180 days from its publication date. According to it, ventilators undergoing registry may, as an exception, present the certificates from the Medical Device Single Audit Program (MDSAP) or from the Brazilian Association of Technical Standards (*Associação Brasileira de Normas Técnicas -* ABNT) NBR ISO 13485:2016,^(34)^ instead of Good Manufacturing Practices certificates, waiving the presentation of a Brazilian System of Compliance Assessment (*Sistema Brasileiro de Avaliação da Conformidade* - SBAC) certificate. This does not mean that ventilator manufacturers and/or developers are exempt to comply with the applicable regulations. Therefore, the development of ventilators requires specific tests to be performed according to ABNT’s criteria. It is strongly recommended carefully reading of the ABNT NBR 60601-1, ABNT NBR ISO 80601-2-12 and ANVISA’s Technical Orientative Note (*Nota Técnica Orientativa*) 001/2020 during the development of such devices (Table 1).^(35-37)^ Additionally, it is recommended to read the ABNT PR 1003:2020 that refer to mechanical ventilators for critical care, containing the applicable requirements and guidance related to safety and performance for the project and manufacturing purposes; its validity matches the RDC 349/2020.^(38)^ This document describes the several types of mechanical ventilators (for critical care, transportation/emergency, sleep apnea, etc.) and makes clear their differences from the manual resuscitator (also known as AMBU) which has no controls, protective systems, or alarms. Besides, the valves system of the AMBU device was developed for short-term manual use, not appropriate for long-term therapy.

**Table 1:** Minimum requirements for electrical safety and performance testing

ABNT NBR IEC 60601-1:2010+Amendment 1:2016 (general rule), item (location)^(35,37)^	ABNT NBR ISO 80601-2-12:2014 (specific rule on critical care ventilators), item (location)^(36,37)^	AMIB (medical technical note)^(39)^
Entry power (4.11) Minimum categorization items (6.2, 6.3 e 6.6) Marking readability and durability (7.1.2/7.1.3) Minimum marks (7.2.2-1st pair.; 7.2.6; 7.2.7; 7.2.9; 7.2.10; 7.2.11; 7.2.12) Control and instrument marks (7.4) User's guide (7.9) Rules for protection against electric shock (8.1) Electric current limits, tension and energy for the accessible parts (8.4.2) Parts separation (8.5) Protective grounding and others (8.6) Leakage current (8.7) Dielectric strength (8.8.3) Portion to the connected to the network, components and layout (8.11) Mobile parts (9.2) Instability (9.4) Parts subject to pneumatic pressure (9.7) Excess temperatures (11.1.1 e 11.1.3) IP Protection grade (11.6.5) Dangerous situations (13.1.2 e 13.1.3) Cooling blockade (13.2.7) Mobile parts locking (13.2.8) Software (14) Push (15.3.2) Impact (15.3.3) Molding stress relief (15.3.6) Connectors construction (15.4.1) Batteries (15.4.3) Indicators (15.4.4)	Overpressure (201.4.11.101.1) Medical device identification, labeling and documents (201.7) Additional aspiration procedure requirements (201.9.101) Leakage (201.11.6.4) Additional water penetration and particulate material requirements - IP 21 (201.11.6.5.101) Power failure technical alarm (201.11.8.101.1) Internal power source (battery) and associated alarm (201.11.8.101.2) Volume control ventilation mode (201.12.1.101) Pressure control ventilation mode (201.12.1.102) Released volume monitoring (201.12.1.103) Oxygen monitor (201.12.4.101) Airway pressure measurement (201.12.4.102) Expired volume measurement (> 50mL), in the same conditions as the trial for item 201.12.1.101 (201.12.4.103.1) Maximal pressure protection device (201.12.4.104) High-pressure protection and alarm device (201.12.4.105) PEEP alarm conditions (201.12.4.106) Obstruction alarm conditions (201.12.4.107) Protection against accidental adjustments (201.12.101) Gas source failure (201.13.102) Software lifecycle (201.14.101) Complete VBS leakage (201.102.7.1) Spontaneous breathing during power source loss (201.103) Delivered oxygen concentration (201.15.102) VBS connectors (201.101.3) VBS requirements and accessories (201.102.1 a 201.102.3) Labeling (201.12.102) Training (201.104)	VCV and/or PCV modes Driving pressure control (above PEEP) on PCV mode (5cmH2O - 30cmH2O) and inspired tidal volume on VCV mode (50mL - 700mL) FiO2 control (21% - 100%) PEEP (0cmH2O - 20cmH2O) Inspiratory time control (PCV mode) in seconds (0.3 to 2.0 seconds) and inspiratory flow (on VCV mode) up to 70 L/minute Respiratory rate control (8rpm a 40rpm) Airway pressure measurement (analogical or digital manometer)* Expired tidal volume measurement, whenever possible; Maximal airway pressure alarm*, leakage and gas source failure High-capacity HEPA filter attachment possibility (N99 or N100) in the expiratory exit If possible, have an at least 2 hours capacity battery

ABNT - *Associação Brasileira de Normas Técnicas*; AMIB - *Associação de Medicina Intensiva Brasileira*; PEEP - positive end-expiratory pressure; VBS - ventilator breathing system; VCV - volume control ventilation; PCV - pressure control ventilation; FiO2 - fraction of inspired oxygen; HEPA - high efficiency particulate arrestance.

* The item "Airway pressure measurement (analogical or digital manometer) allows the use of an analogical manometer, however this has an impact on the requirements for the item "Maximal airway pressure alarm, leakage and gas source failure" on the use of a maximal airway pressure alarm. The use of a pressure alarm would be hardly possible if an analogical manometer is adopted, as it is much more feasible by means of a digital manometer.

It should be stressed that, although RDC 349/2020 exceptionally exempt the products of presenting an SBAC certification, this RDC does not exempt products’ manufacturers, importers, and developers from the safety and efficacy requirements according to RDC 56/2001, in addition to other applicable regulations.

Additional to the above described technical rules and regulatory requirements described below, in April 2020 the Brazilian Association of Intensive Medicine (*Associação de Medicina Intensiva Brasileira* - AMIB) published a technical medical note regarding the features of mechanical ventilators used in COVID-19 patients.^(39)^ In this note, AMIB emphasizes that the requirements were decided for an exceptional time due to the large number of patients requiring mechanical ventilation, and should be met regardless of the type of ventilator.^(39)^ The minimum requirements are shown in table 1.

## VENTILATORS RESEARCH AND DEVELOPMENT

Another challenge for developers is the lack of knowledge regarding the minimum test requirements for early research and development project phases. Currently, creative procedures and agile methodologies are widespread in product development processes, aiming to speed-up the initial prototype validation. It is important to emphasize that the results from this phase tests should be part of the required regulatory documents to start the ANVISA registration procedure and is fundamental that these tests comply with the current regulations.

### Preclinical bench-testing

The developing ventilator should undergo several tests designed to check the basic safety and performance requirements. In addition to operation and safety-related tests, the performance should be tested in a lung simulator, with different load adjustments, allowing the simulation of close to real-life lung features, with different capacities, resistances, and compliances.

The lung testing device provided with commercial mechanical ventilators is only suitable for bedside ventilator testing, before its connection to the patient. This lung testing device is not appropriate to assess performance related to volume, flow, pressures, and inspiratory and expiratory times. This is one of the issues for the development of alternative ventilators, as the lung simulator algorithm should be adaptative, to provide correct ventilation in real-life lungs, which, in addition to their dynamically changing characteristics of compliance and resistance during the different breaths, may also show a non-linear behavior.

Fundamentally, all tested variables (TV, respiratory flow, inspiratory pressure, P_plateau_, PEEP, inspiratory time, expiratory time) are recorded for the different evaluated scenarios. Also, the FiO_2_ provided by the gas mixer should be measured.

### Preclinical animal studies

After validation of minimum requirements according to the safety and performance technical rules, the next step refers to animal model testing. Autopsies of patients that have died from COVID-19 evidenced diffuse alveolar injury, characterized by the alveolar formation of hyaline membrane, inflammatory cell accumulation, interstitial congestion due to edema, and fibroblast proliferation in organization phase.^(40,41)^ Preclinical animal tests using healthy lungs are not likely to reflect the behavior seen in patient’s lungs with SARS-CoV-2 infection while testing experimental ventilators. Therefore, it is fundamental that tests are conducted in validated acute lung injury animal models.

There are several animal models described in the literature.^(42,43)^ The majority is based on the clinical disorders associated with acute respiratory distress syndrome (ARDS) in humans.^(43)^ Unfortunately, no animal model of ARDS can accurately replicate the complex pathophysiological changes seen in patients with such disease.^(44)^ Models presenting good reproducibility are based in porcine and ovine^(43,45)^ after lipopolysaccharide (LPS) intravenous injections,^(45)^ total surfactant lavage followed by aggressive mechanical ventilation (using high volumes and pressures),^(46)^ intravenous oleic acid injection,^(45)^ or intratracheal hydrochloric acid administration.^(47)^

It is expected that alternative ventilators provide appropriate PEEP levels to recruit collapsed alveoli, adequate tidal volumes and inspiratory pressures to protect the lungs from worsening the established injuries, and to be able to maintain satisfactory gas exchange during long-term mechanical ventilation. It is of upmost importance that all preclinical animal tests should document properly the adjusted ventilatory settings, vital signs, and arterial gasometry results, to set up a dossier for ANVISA registration. Additionally, it should be stressed that every research should be conducted under the appropriate regulations involving the care of the test animals and mandatory authorization from an Animal Research Ethics Committee should be granted.

### Clinical trials

A clinical trial is a systematic investigation to test a hypothesis, involving human participants. Different clinical trials methodologies can be used. Observational studies assess patients under routine medical care, while clinical trials test an intervention and evaluate the intervention’s safety and/or effectiveness. Each clinical trial has a Clinical Investigation Plan (more details can be found in the session ‘Clinical trial dossier submission’). Before starting the clinical trial, the research protocol should be previously approved by the Research Ethics Committee, and in the case of multicenter clinical trials, by the National Research Ethics Committee (*Comissão Nacional de Ética em Pesquisa* - CONEP), submitted via Plataforma Brasil (an electronic platform for Research Ethics submission in Brazil).^(48)^

Every participant should sign an Informed Consent Form before any study-related procedure is conducted. Any clinical trial should rigorously comply with the Good Clinical Practices.^(49)^

## REGULATORY PROCESS PHASES

The health-related regulatory process for medical devices aims the application of technical and regulatory requirements for the entire product life-cycle, either pre- or postmarketing.

Therefore, the regulation starts from checking the good manufacturing practices requirements, from its concept, project verification and validation stages, product risk-management, clinical validation, trackability, preventive and corrective technical assistance, technovigilance, deactivation, and disposal.

All procedures cited above are organic and receive constant feedback from those accountable for the device’s development, manufacturing, and availability to the end-user. Legally, the company’s Legal Responsible and the Technical Responsible are accountable for compliance with technical and legal requirements.

**Figure 6:**
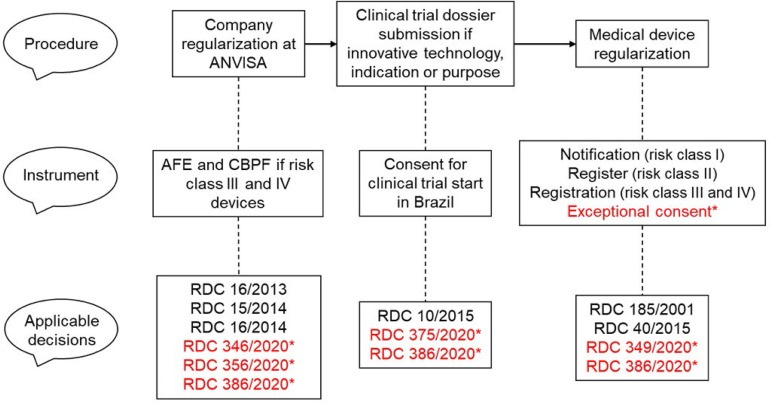
Steps for medical devices premarketing regularization. ANVISA - *Agência Nacional de Vigilância Sanitária;* AFE *- Autorização de Funcionamento de Empresa;* CBPF - *Certificação de Boas Práticas de Fabricação*; RDC - *Resolution of the Collegiate Board*. * Extraordinary and temporary measures to deal with the public health emergency related to acute severe respiratory syndrome coronavirus 2.

The premarketing stages chronology involving submission requirements to ANVISA are displayed in figure 6. Extraordinary and temporary administrative acts related to the COVID-19 pandemics are highlighted.

### Regularization of the company at ANVISA

Before registering any device at ANVISA, the responsible company should be regularized at the local Sanitary Surveillance (VISA), which may conduct inspections to check on technical and operational aspects and issue a business license, and at ANVISA to obtain a Company Operating Authorization (*Autorização de Funcionamento de Empresa* - AFE) for manufacturing healthcare products, under the RDC 16/2014.

In addition, for companies manufacturing high- and maximal-risk devices (classes III and IV), RDC 15/2014 requires the obtention of a Good Manufacturing Practices Certificate (*Certificação de Boas Práticas de Fabricação* - CBPF) issued by ANVISA.

### Medical device registration

RDC 185/2001 is related to medical device registration, including risk III class, as critical care and emergency and transport ventilators are rated. Registration of these devices should be requested to ANVISA with the documents and information required in RDC 185/2001. ANVISA’s technical staff evaluates the process and decides on approval; when necessary they can request additional information and documents. For the agility of the process, it is important that the registration rules are appropriately interpreted. To render this process easier ANVISA provided a complete manual for medical device manufacturers and importers.^(50)^ This manual has overall information on the submission process and a detailed description of the registration workflow, as briefly described below:^(50)^

- Step 1 - manufacturer regularization at the VISA: obtaining the AFE from ANVISA, and an Operating License (OL) at the city or state VISA (also known as Operating Permit - OP). Also, the manufacturing company must comply with Good Manufacturing Practices.
- Step 2 - device’s sanitary identification: corresponds to its identification and categorization (according to the risk and objective). It is also checked if the device requires complementary certifications and reports, registration or notification, e.g. sanitary identification or economic information report.
- Step 3 - request identification: if the device is required to be registered or to be notified to ANVISA.
- Step 4 - electronic submission: the action that effectively starts the medical device registration process at ANVISA.
- Step 5 - protocol submission.

### Decisions issued in the pandemic context

The manual provided by ANVISA regarding medical device registration processes^(50)^ fails to include all the most recent RDCs related to medical devices and the SARS-CoV-2 virus pandemic. Below we present a brief description of each normative change issued since the pandemic started, in March 2020.

- ANVISA RDC 346/2020: establishes criteria and extraordinary/temporary procedures for good manufacturing practices certification for registration issues and post-registration changes for pharmaceutic supplies, drug products, and healthcare products related to the international public health emergency due to the new coronavirus.
- ANVISA RDC 349/2020: establishes extraordinary and temporary procedures for dealing with personal protective equipment regularization requests, for mechanical ventilators and other medical devices identified by ANVISA as strategic, due to the international public health emergency related to the new coronavirus. Issued on the Official Union Journal (*Diário Oficial da União* - DOU) number 55, on March 20^th^, 2020.
- ANVISA RDC 356/2020: establishes extraordinary and temporary requirements for manufacturing, importing, and buying priority medical devices for healthcare services, regarding the international public health emergency due to the SARS-CoV-2 virus pandemic. Republished on extra DOU number 57-C on March 24^th^, 2020, and DOU number 62 on March 31^st^, 2020.
- ANVISA RDC 386/2020: establishes extraordinary and temporary criteria and procedures for Exceptional Consent for Manufacture, Commercialization, and Donation of Emergency and Transitory Respiratory Support type “Automatized AMBU” devices; issued on DOU number 92-B on May 15^th^, 2020.

### Clinical trial dossier submission

It is important to highlight that, in addition to the ANVISA regularization of the medical device, a clinical assessment is required, using clinical trials in cases when the used technology or device purpose is innovative. Innovative is considered, e.g. the device is adapted to add a new not previously existing function or inclusion of a new indication for a previously marketed device.

In April 2020, the RDC 375/2020 was issued regarding the submission of clinical trials designed to validate class III and IV medical devices. Those class of devices were identified as health services priority related to the international public health emergency related to the SARS-CoV-2 virus. Clinical trials meeting these criteria can be submitted as a clinical trial notification. This clinical trial notification should comprise the following documents:^(51)^

a) Completed presentation form - available at the ANVISA website.^(51)^
b) Payment voucher, or tax exemption proof.
c) Clinical trial protocol, compliant with Good Clinical Practices.^(49)^
d) Proof or the trial registration at the International Clinical Trials Registration Platform (ICTRP) or other site accepted by the International Committee of Medical Journals Editors (ICMJE).
e) Ethics Committee approval for the first clinical trial site submitting the protocol.

**Figure 7:**
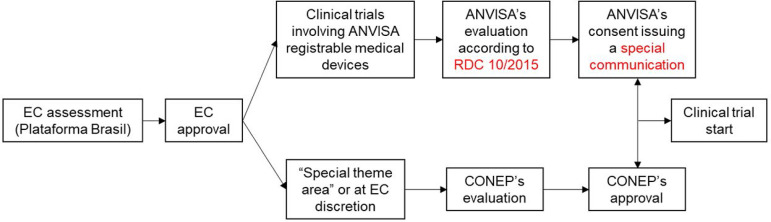
Clinical trials submission rite to Ethics Committee, National Ethics Committee, and *Agência Nacional de Vigilância Sanitária.* EC - Ethics Committee; ANVISA - *Agência Nacional de Vigilância Sanitária;* RDC - Resolution of the Collegiate Board; CONEP - *Comissão Nacional de Ética em Pesquisa*.

Source: Adapted from: Brasil. Agência Nacional de Vigilância Sanitária. Perguntas e Respostas RDC 10/2015. V. 3. Brasília: Anvisa; 2019. [citado 2020 Maio 15Σ. Disponível em: http://portal.anvisa.gov.br/documents/33912/264679/Perguntas+e+Respostas+-+RDC+10-2015+docx.pdf/2bad2269-5c0a-4cbe-b6b1-78db1861c5cc.^(52)^

ANVISA should provide previous clinical trial consent, considering that it will serve for future registration of a medical device or changing the originally registered clinical indications. Therefore, ANVISA should assess the trial methodological issues, regarding the ability to support safety and efficacy evidence as well as Good Clinical Practice issues related to the clinical trial. The clinical trial submission flow should follow the sequence displayed in figure 7.^(52)^

It should be stressed that, regarding the COVID-19 pandemic, changes may occur in these regulatory settings, and updated information is available on the ANVISA website.

## CONCLUSION

Mechanical ventilation is much more than just pumping air into patients’ lungs. Several precise and simultaneous adjustments are required, on the airway pressures, tidal volume, the fraction of inspired oxygen, respiratory rate, I:E ratio, among others. Developing a low-cost and quick manufacturing ventilator able to meet these requirements is not an easy task. Medical device development and research require scientifically validated methods to be followed.

The challenges are increased by the regulatory requirements. A genuine effort from the regulatory authorities is in place to reduce the requirements and speed up the evaluation process, however, the safety exigences can’t be waived in the best interest of the population.

However, managers, front line healthcare professionals and the population are confident that some (or several) of the ongoing projects will meet the required technical specifications and will, soon, contribute to avoid that more lives are lost in this pandemic.
